# Clinical characteristics of adrenal crisis in 371 adult patients with glucocorticoid-induced adrenal insufficiency

**DOI:** 10.3389/fendo.2024.1510433

**Published:** 2024-12-17

**Authors:** Ying Qiu, Ying Luo, Xinqian Geng, Yujian Li, Yunhua Feng, Ying Yang

**Affiliations:** ^1^ Department of Endocrinology, The Affiliated Hospital of Yunnan University, Kunming, China; ^2^ School of Medical, Kunming Medical University, Kunming, China; ^3^ School of Statistics, Beijing Normal University, Beijing, China

**Keywords:** GIAI, adrenal crisis, clinical characteristics, prediction models, neutrophil-lymphocyte ratio, eosinophil-lymphocyte ratio

## Abstract

**Background:**

Glucocorticoid-induced adrenal insufficiency (GIAI) is a hypothalamic-pituitary-adrenal (HPA) axis dysfunction caused by long-term use of exogenous steroids. Adrenal crisis (AC) is an acute complication of GIAI and one of the reasons for the increased risk of death. This study aims to analyze the clinical characteristics of GIAI patients with AC and explore the related risk factors.

**Methods:**

Clinical data of adult GIAI patients treated at our hospital between January 1, 2014, and December 31, 2023 were included. The demographic characteristics, clinical characteristics, laboratory tests and comorbidities of the patients were collected. Univariate and multivariate regression analyses were used to explore the variables related to the occurrence of AC, and prediction models were constructed.

**Results:**

51 patients (13.75%) developed AC during hospitalization. Mortality was significantly higher in patients with AC than in those without AC. Multivariate logistic regression analysis showed that infection, psychiatric symptoms, serum sodium, albumin, neutrophil-lymphocyte ratio (NLR) and eosinophil-lymphocyte ratio (ELR) were independent risk factors for AC. Among the prediction models constructed by machine learning algorithms, logistic regression model had the best prediction effect.

**Conclusion:**

This study investigated the clinical characteristics of AC in GIAI patients. NLR and ELR may be effective predictors of AC in GIAI patients, and combined with other clinically significant indicators, an effective prediction model was constructed. Logistic regression model had the best performance in predicting AC in GIAI patients.

## 1 Introduction

Glucocorticoids (GCs) are widely utilized in clinical practice for their anti-inflammatory and immunosuppressive effects (1). GCs are administered through various routes (oral, inhaled, intranasal, intra-articular, topical, intravenous) and are often over extended periods (2). When administered at doses exceeding the equivalent of 5 mg prednisone and used continuously for three weeks or more, they can suppress adrenal function, leading to glucocorticoid-induced adrenal insufficiency (GIAI) (2). GIAI, the most common cause of secondary adrenal insufficiency (SAI), results from the long-term suppression of the hypothalamic-pituitary-adrenal (HPA) axis due to exogenous steroid use (3). Prolonged exposure to high cortisol levels in GIAI can lead to numerous adverse metabolic and cardiovascular effects, such as significant alterations in body composition (obesity, muscle wasting, osteoporosis), neuropsychiatric disorders (cognitive deficits, depression, sleep disturbances), metabolic syndrome (obesity, hypertension, insulin resistance, and dyslipidemia), hypercoagulable states, and immunosuppression (4–7). These complications not only diminish patients’ quality of life but also pose long-term health challenges and serious health risks, including adrenal crisis (AC) or severe gastrointestinal bleeding, which can be fatal in extreme cases (8).

AC is an acute physiological disorder that occurs when circulating levels of adrenal steroid hormones are insufficient to meet physiological needs representing a serious and potentially life-threatening complication of adrenal insufficiency (AI) (9, 10). The incidence of SAI is approximately twice that of primary adrenal insufficiency (PAI), yet the incidence of AC is higher in PAI (11). AC is relatively rare in patients receiving GCs for other health issues, possibly due to some residual adrenocortical function or incomplete HPA axis inhibition (12), but its potential to threaten life cannot be ignored. There is no universally accepted definition of AC, leading to variations among clinicians and studies (13). Simple patient education is insufficient to prevent many cases of AI, making AC prevention a critical issue in endocrinology with uncertain strategies for reducing its incidence. Age and severe comorbidities are suggested as risk factors for AC in AI patients, though the mechanisms are unclear (14). Physiological stressors, particularly infection, are the most common triggers of AC, and further research is needed to identify inflammatory markers at AC presentation. Continuous monitoring of AC events is crucial for prevention. The aim of this study is to analyze the clinical characteristics of AC in adult patients with GIAI and to construct related risk prediction models, providing a new perspective for future research into the prevention of AC, thereby reducing the incidence of AI events with a view to improving the long-term prognosis and quality of life of patients.

## 2 Methods

### 2.1 Research design and ethics

This retrospective study was reviewed and approved by the Ethics Committee of the Affiliated Hospital of Yunnan University, China (Ethics Approval No. 2024031), and the institutional review committee waived the written informed consent.

### 2.2 Data acquisition

This study included 371 patients diagnosed with GIAI at the Affiliated Hospital of Yunnan University between January 1, 2014, and December 31, 2023. The general information of the patients collected at the time of admission is shown in **Table 1**.

**Table 1:** Variables included in this study.

Categories of variables	Specific variable values
**Demographic measures**	Age, gender (male or female), BMI, occupation (civil servant, retired, unemployed, skilled, etc.), death
**Hospitalization characteristics**	Medical payment method (basic medical insurance for urban workers, basic medical insurance for urban residents, new rural cooperative medical care, self-pay, other), length of stay, reasons for glucocorticoid use and types of glucocorticoid use
**Clinical characteristic**	Hypertension, diabetes, digestive diseases, osteoporosis, infection, psychiatric symptoms, eye diseases, moon-like facies by glucocorticoid
**Laboratory finding**	Blood glucose, blood potassium, blood sodium, blood calcium, red blood cell count, hemoglobin count, white blood cell count, neutrophil count, lymphocyte count, eosinophilic count, platelet count, total bilirubin, aspartate aminotransferase, alanine aminotransferase, albumin, globulin, uric acid, serum creatinine, neutrophil-to-lymphocyte ratio, eosinophil-to-lymphocyte ratio, platelet-to-lymphocyte ratio, sodium-to-potassium ratio.

BMI, Body mass index.

The inclusion criteria included: (1) diagnosis between January 1, 2014, and December 31, 2023; (2) all cases referred according to the diagnostic criteria recommended by the European Society of Endocrinology and Endocrine Society Joint Clinical Guideline (1): (i) measurement of morning serum cortisol levels suggested HPA axis dysfunction or; (ii) presenting with signs and symptoms of AI following current or recent use of non-oral glucocorticoid medications, or; (iii) concomitant use of multiple glucocorticoid preparations, or; (iv) use of high-dose inhaled or topical glucocorticoids, or; (v) use of inhaled or topical glucocorticoids for > 1 year, or; (vi) received intra-articular glucocorticoid injections within the last 2 months, or; (vii) Patients exhibiting signs and symptoms of exogenous Cushing’s syndrome following current or prior glucocorticoid therapy suggest a diagnosis of GIAI.

The exclusion criteria included: (1) younger than 18 years; (2) PAI; (3) incomplete data collection; (4) hospital stay <1 day.

### 2.3 Statistical analysis

#### 2.3.1 Risk factor analysis

The data collected were analyzed using IBM SPSS Statistics for Windows V25.0 software. The Kolmogorov-Smirnov test was used to assess the normality of continuous variables. An independent sample t-test was used for normally distributed measurement data, expressed as mean ± standard deviation (SD); Mann-Whitney test was used as a non-parametric test for non-normally distributed measurement data, expressed as median plus IQR. For categorical variables, the chi-square test or Fisher exact test was used and expressed as frequencies and percentages. The statistically significant indicators in univariate analysis combined with clinically significant indicators were first screened using univariate logistic regression. Multivariate logistic regression was performed for identifying significant risk factors. All results with a two-sided p value of less than 0.05 were considered to indicate statistical significance.

#### 2.3.2 Prognostic model construction

All subjects were randomly divided into two parts, the training set and the validation set, in a ratio of 7:3, using R software. Logistic regression, decision tree, random forest and SVM algorithms in R software were used to fit the model of the training set data, and the test set data were used for prediction and discrimination. Additionally, the accuracy of the decision tree model could be optimized by pruning the decision tree and combining multiple decision trees using the random forest method. Thus, the pruned decision tree model was included in the overall results for comparison with the random forest model. The precision, recall, F1 score and accuracy of each model were comprehensively compared. Additionally, the receiver operating characteristic (ROC) curve was plotted for each model, and the area under the ROC curve (AUC) was calculated to evaluate the predictive performance of the binary classification models.

## 3 Results

### 3.1 Baseline information

We collected the clinical data of 602 patients with adrenal disease who were treated in our hospital between January 1, 2014 and December 31, 2023. After considering the inclusion and exclusion criteria, a total of 371 GIAI patients were included in this study. 51 GIAI patients (13.75%) were diagnosed with AC during hospitalization, and 320 patients (86.25%) were not diagnosed with AC during hospitalization (**Table 2**). In terms of demographic and hospitalization characteristics, the mortality rate of patients with AC (62.75%) was significantly higher than that of patients without AC (44.06%), and the difference was statistically significant (**Table 2**).

**Table 2:** Demographic characteristics and hospitalization characteristics of GIAI patients.

Patient characteristics	Adrenal Crisis	No Adrenal Crisis		Z/χ^2^ value	P
**Total (n= number)**	51(13.75%)	320(86.25%)			
**Age**	54.50 (48.28,70.46)	59.33 (48.45,68.94)		-0.553	0.580
Age group					
** 18.00-29.99**	1(1.96%)	11(3.44%)		4.230	0.646
** 30.00-39.99**	4(7.84%)	21(6.56%)			
** 40.00-49.99**	12(23.53%)	58(18.13%)			
** 50.00-59.99**	11(21.57%)	73(22.81%)			
** 60.00-69.99**	9(17.65%)	88(27.50%)			
** 70.00-79.99**	10(19.61%)	56(17.5%)			
** ≥80.00**	4(7.84%)	13(4.06%)			
Gender					
** Male**	33(64.71%)	177(55.31%)		1.580	0.209
** Female**	18(35.29%)	143(44.29%)			
**BMI**	24.00(21.00,28.00)	25.00(22.00,28.00)		-1.672	0.094
Occupations					
** Farmer**	23(45.10%)	116(36.25%)		1.476	0.688
** Retirees**	10(19.61%)	73(22.81%)			
** Unemployed**	4(7.84%)	28(8.75%)			
** Others**	14(27.45%)	103(32.19%)			
**Average length of stay**	11.00(8.00,13.00)	10.00(7.00,14.50)		-0.006	0.996
Medical payment methods					
** Basic medical insurance for urban residents**	34(66.67%)	145(45.31%)		11.710	**0.020**
** Basic medical insurance for urban employees**	8(15.69%)	107(33.44%)			
** The new rural cooperative medical system**	3(5.88%)	39(12.19%)			
** Self-pay**	5(9.80%)	18(5.63%)			
** Others**	1(1.96%)	11(3.44%)			
Reasons for GC use					
** Gout**	22(43.14%)	122(38.1%)		0.906	0.824
** Rheumatoid arthritis**	6(11.76%)	50(15.6%)			
** Pain**	6(11.76%)	45(14.1%)			
** Others**	17(33.33%)	103(32.2%)			
Types of GC Used					
** Dexamethasone**	18(35.29%)	83(25.9%)		1.944	0.378
** Prednisone tablets**	7(13.73%)	50(15.6%)			
** Others**	26(50.98%)	187(58.4%)			
**Died (%)**	32(62.75%)	141(44.06%)		6.170	**0.013**

GC, glucocorticoid; BMI, Body mass index.

Bold values mean P values < 0.05.

In terms of clinical characteristics, chi-square test results revealed significant differences in diabetes (P=0.005), infection (P < 0.001), osteoporosis (P=0.013), and psychiatric symptoms (anxiety and depression) (P < 0.001) between AC and non-AC patients. However, no significant differences were observed in hypertension, glucocorticoid-induced full moon face, digestive system diseases (including gastrointestinal bleeding, ulcers, etc.), and eye diseases (including glucocorticoid-induced glaucoma, retinopathy, etc.) between AC and non-AC patients (**Table 3**).

**Table 3:** Clinical characteristics of GIAI patients.

Variable		Adrenal Crisis (n=51)	No Adrenal Crisis (n=320)	χ^2^ value	P
**Diabetes**	Yes	38(74.51%)	171(53.44%)	7.941	**0.005**
	No	13(25.49%)	149(46.56%)		
**Hypertension**	Yes	31(60.78%)	169(52.81%)	1.329	0.515
	No	20(39.22%)	151(47.19%)		
**Moon-like facies by glucocorticoid**	Yes	37(72.55%)	218(68.13%)	0.401	0.527
	No	14(27.45%)	102(31.87%)		
**Infection**	Yes	47(92.16%)	201(62.81%)	17.092	**<0.001**
	No	4(7.84%)	119(37.19%)		
**Digestive system diseases**	Yes	26(50.98%)	138(43.13%)	1.101	0.294
	No	25(49.02%)	182(56.88%)		
**Osteoporosis**	Yes	42(82.35%)	207(64.69%)	6.220	**0.013**
	No	9(17.65%)	113(35.31%)		
**Psychiatric symptoms**	Yes	10(19.61%)	18(5.63%)	12.326	**<0.001**
	No	41(80.39%)	302(94.38%)		
**Eye diseases**	Yes	10(19.61%)	69(21.56%)	0.100	0.751
	No	41(80.39%)	251(78.44%)		

Bold values mean P values < 0.05.

From the laboratory examination, non-parametric test results indicated that blood glucose (P < 0.001), white blood cell count (P < 0.001), neutrophil count (P < 0.001) and neutrophil-lymphocyte ratio (NLR) (P < 0.001) were significantly higher in AC patients compared to non-AC patients. Serum sodium (P < 0.001), erythrocyte count (P=0.009), hemoglobin count (P=0.004), eosinophil count (P < 0.001), lymphocyte count (P=0.001), albumin count (P=0.037), and eosinophil-lymphocyte ratio (ELR) (P < 0. 001) were significantly lower in AC patients compared to non-AC patients (**Table 4**).

**Table 4:** Laboratory findings of GIAI patients.

variables	Adrenal Crisis (n=51)	No Adrenal Crisis (n=320)	Z value	P
**Blood glucose**	9.00(6.21,15.15)	6.74(4.84,10.38)	-3.513	**<0.001**
**Blood potassium**	3.57(3.30,3.90)	3.51(3.22,4.00)	-0.121	0.904
**Blood sodium**	131.00(126.60,137.10)	139.10(135.80,141.60)	-6.191	**<0.001**
**Blood calcium**	2.13(2.04,2.28)	2.11(2.00,2.26)	-1.064	0.287
**Red cell count**	3.93(2.96,4.58)	4.29(3.69,4.69)	-2.631	**0.009**
**Hemoglobin count**	119.00(89.00,137.00)	131.00(110.00,144.00)	-2.873	**0.004**
**White cell count**	14.02(10.30,17.44)	9.03(7.17,11.30)	-5.686	**<0.001**
**Neutrophil count**	13.05(9.42,16.14)	6.29(4.73,8.23)	-8.209	**<0.001**
**Eosinophil count**	0.02(0.00,0.04)	0.06(0.02,0.14)	-6.045	**<0.001**
**Lymphocyte count**	1.23(0.74,1.73)	1.62(1.04,2.17)	-3.210	**0.001**
**Platelet Count**	236(155,324)	224.00(176.25,300.00)	-0.160	0.873
**Total bilirubin**	10.10(6.50,16.10)	10.75(7.90,15.33)	-0.700	0.484
**Aspartate aminotransferase**	20.00(14.00,38.00)	20.00(16.00,29.00)	-0.267	0.789
**Alanine aminotransferase**	21.00(11.00,36.00)	23.00(15.00,36.00)	-1.477	0.140
**Albumin**	32.60(28.20,37.40)	34.50(30.70,39.40)	-2.084	**0.037**
**Globulin**	23.90(22.00,26.30)	24.50(20.73,27.88)	-0.529	0.597
**Uric acid**	412.00(284.00,509.00)	411.50(303.75,518.75)	-0.153	0.879
**Serum creatinine**	67.00 (53.00,111.00)	70.00(53.00,94.75)	-0.113	0.910
**NLR**	8.80(5.44,20.14)	3.87(2.33,6.50)	-7.226	**<0.001**
**ELR**	0.01(0.00,0.04)	0.04(0.02,0.09)	-5.404	**<0.001**
**PLR**	190.17(111.80,279.59)	141.04(97.81,221.86)	-2.403	**0.016**
**BMR**	1331.67(1194.38,1534.16)	1352.37(1217.16,1532.19)	-0.772	0.440
**Na-to-K ratio**	37.01(32.58,41.00)	39.17(34.26,43.26)	-1.823	0.068

NLR, Neutrophil-to-lymphocyte ratio; ELR, Eosinophil-to-lymphocyte ratio; PLR, Platelet-to-lymphocyte ratio; Na-to-K ratio, Sodium-to-Potassium; BMR, Basal metabolic rate.

Bold values mean P values < 0.05.

### 3.2 Results of logistic regression analysis

Univariate logistic regression analysis showed that diabetes, infection, osteoporosis, psychiatric symptoms, blood glucose, blood sodium, erythrocyte count, hemoglobin count, white blood cell count, neutrophil count, eosinophil count, lymphocyte count, albumin, NLR and ELR were risk factors for AC (**Table 5**). Multivariate logistic regression analysis of the identified important risk factors showed that infection, psychiatric symptoms, serum sodium, albumin, NLR and ELR were independent risk factors for AC (**Table 6**).

**Table 5:** Results of one-way logistic regression analysis.

variables	β	SE	Wald χ^2^	P	OR	95%CI
**Diabetes**	0.935	0.340	7.548	0.006	2.547	(1.307,4.962)
**Infection**	1.940	0.534	13.217	<0.001	6.956	(2.445,19.794)
**Osteoporosis**	0.935	0.385	5.884	0.015	2.548	(1.197,5.423)
**Psychiatric symptoms**	1.409	0.428	10.834	0.001	4.092	(1.768.9.470)
**Blood glucose**	0.074	0.023	10.178	0.001	1.077	(1.029,1,128)
**Blood sodium**	-0.167	0.027	37.695	<0.001	0.846	(0.803,0.893)
**Red cell count**	-0.586	0.184	10.177	0.001	0.557	(0.388,0.798)
**Hemoglobin count**	-0.018	0.006	10.850	0.001	0.982	(0.971,0.993)
**White cell count**	0.201	0.034	34.539	<0.001	1.222	(1.143,1.307)
**Neutrophil count**	0.276	0.039	49.284	<0.001	1.317	(1.220,1.423)
**Eosinophil count**	-17.132	4.154	17.007	<0.001	0.000	(0.000,0.000)
**Lymphocyte count**	-0.721	0.224	10.399	0.001	0.486	(0.314,0.754)
**Albumin**	-0.048	0.024	4.123	0.042	0.953	(0.910,0.998)
**NLR**	0.023	0.011	4.430	0.035	1.024	(1.002,1.046)
**ELR**	-21.736	5.674	14.677	0.000	0.000	(0.000,0.000)
**PLR**	0.000	0.000	0.044	0.834	1.000	(1.000,1.000)

NLR, Neutrophil-to-lymphocyte ratio; ELR, Eosinophil-to-lymphocyte ratio; PLR, Platelet-to-lymphocyte ratio.

**Table 6:** Results of multivariate Logistic regression analysis.

variables	β	SE	Wald X^2^	P	OR	95%CI
**Diabetes**	0.705	0.584	1.460	0.227	2.024	(0.645,6.355)
**Infection**	3.413	1.044	10.695	0.001	30.367	(3.926,234.868)
**Osteoporosis**	1.117	0.571	3.829	0.050	3.057	(0.998,9.363)
**Psychiatric symptoms**	2.349	0.676	12.086	0.001	10.472	(2.786.39.361)
**Blood glucose**	-0.050	0.049	1.021	0.312	0.952	(0.865,1,048)
**Blood sodium**	-0.215	0.040	28.432	<0.001	0.806	(0.745,0.873)
**Hemoglobin count**	-0.018	0.009	3.577	0.059	0.982	(0.964,1.001)
**Albumin**	0.088	0.035	6.405	0.011	1.092	(1.020,1.170)
**NLR**	0.133	0.038	12.495	<0.001	1.143	(1.061,1.230)
**ELR**	-22.136	6.580	11.842	0.001	0.000	(0.000,0.000)

NLR, Neutrophil-to-lymphocyte ratio; ELR, Eosinophil-to-lymphocyte ratio.

### 3.3 Prognosis prediction model

**Figure 1:**
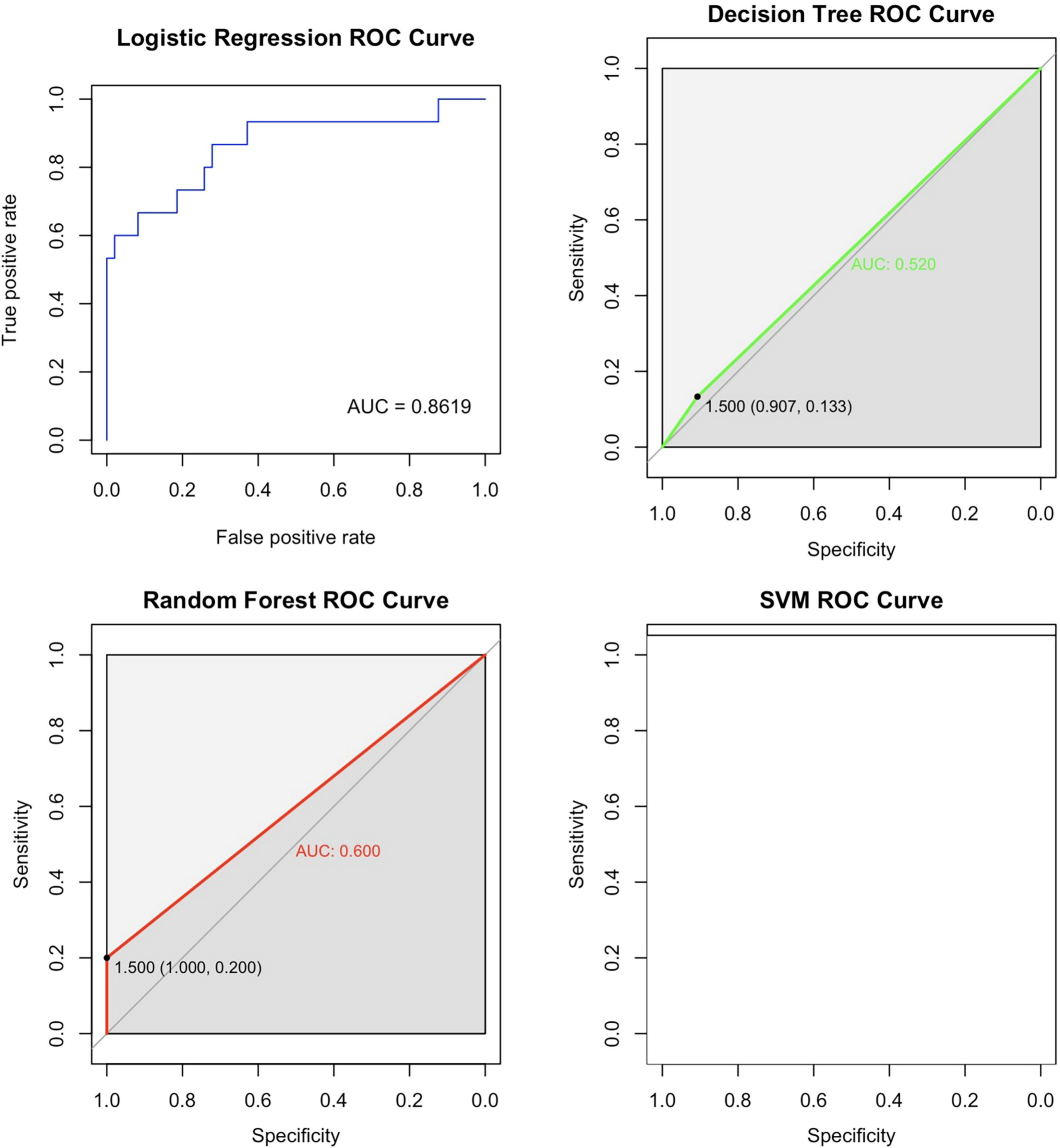
Decision tree. This decision tree predicts the occurrence of AC through a series of tests based on indicators such as neutrophils, albumin, eosinophils, infections, psychiatric disorders, with each node representing a test, branches representing test results, and leaf nodes representing the final classification. The tree displays thresholds for neutrophils, albumin, eosinophils, and infections, and each leaf node also provides sample numbers and accuracy for classification.

The information collected from 371 patients was used to select 33 variables of interest from 44 variables to construct a full logistic model. Considering stepwise regression, non-significant variables were removed and the model was reconstructed using the Akaike Information Criterion (AIC) minimum criterion to obtain a new logistic model to ensure the minimum AIC. Full moon face, infection, psychiatric symptoms, ALT, AST, NLR and ELR were included in the final prediction model. Variables were selected for the decision tree, random forest, and SVM algorithms based on the standards. The decision tree model was constructed to obtain the discrimination criteria shown in **Figure 1** (15, 16).

After constructing the logistic model, decision tree, pruned decision tree and random forest models, the overall precision, recall rate, F1 score and accuracy of the five models are obtained as shown in **Table 7** below are obtained. From the results, in terms of accuracy, all five models exceed 80%, with the Logistic model and random forest model showing relatively high accuracy at 92.86% and 89.29%, respectively. In terms of both accuracy and F1 score, the logistic model performed significantly better than the other models. The recall rates of logistic model, random forest model and SVM model were all 1, indicating almost complete recall. However, the decision tree model had a recall rate of only 18.18%, which is considered low. Overall, the logistic model performed the best and was the most suitable for AC prediction in the GIAI patient dataset presented here.

**Table 7:** Accuracy results of each prediction model.

Prediction model	Precision	Recall rate	F1 score	Accuracy rate
Logistic model	0.4667	1.000	0.6364	0.9286
Decision tree	0.1333	0.1818	0.1538	0.8036
Pruned decision tree	0.1333	0.1818	0.1538	0.8036
Random forest	0.2000	1.000	0.3333	0.8929
SVM model	0.0667	1.0003	0.1250	0.8750

**Figure 2:**
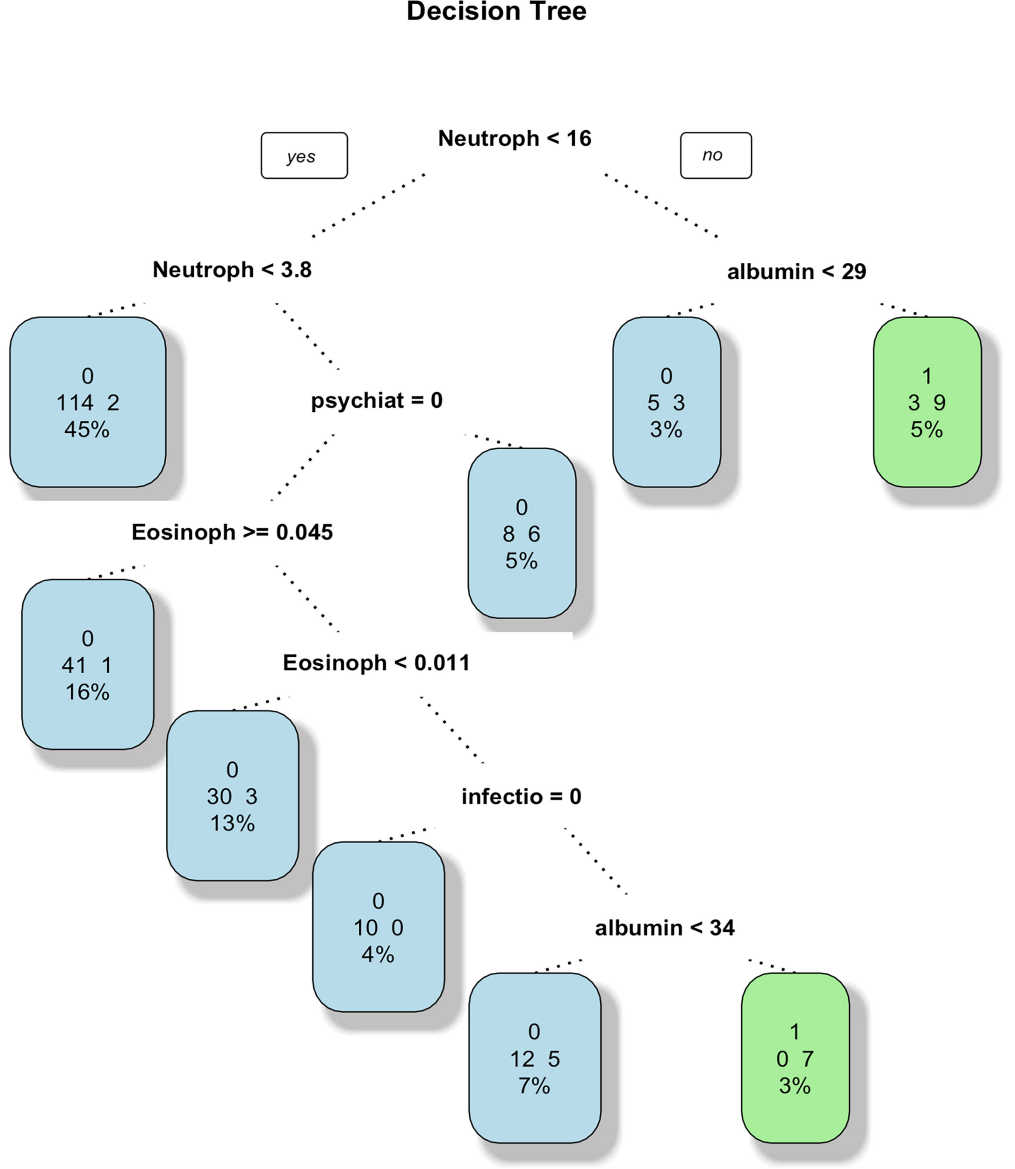
ROC curves for the four models. This ROC curve shows the performance comparison of four machine learning models (logistic regression, random forest, decision tree and support vector machine) on the binary classification problem, and the model accuracy is evaluated by the relationship between the true rate and the false positive rate. The logistic regression model had the best performance with an AUC value of 0.8619. The second is the random forest model, and the SVM model is not an optional model for data prediction in this paper.

In order to further analyze the predictive performance of each model and compare their advantages and disadvantages in predicting AC in GIAI patients, the AUC of each model was calculated and the ROC curve was drawn as shown in **Figure 2** below. Since the predictive effect of pruned decision trees is similar to that of unpruned decision trees, the unpruned model is excluded from this analysis. The ROC curve and AUC value in **Figure 2** show that the logistic model outperforms the other three models, with an AUC value of 0.8619. Considering the accuracy results from the classification and prediction of the models discussed, the logistic model appears to have the best predictive performance for datasets similar to this study. The random forest model ranks second, while the SVM model is not considered a suitable option for data prediction in this study. The logistic and random forest models demonstrated the best predictive performance in this dataset.

### 3.4 Correlation analysis of NLR and ELR with other indicators

**Figure 3:**
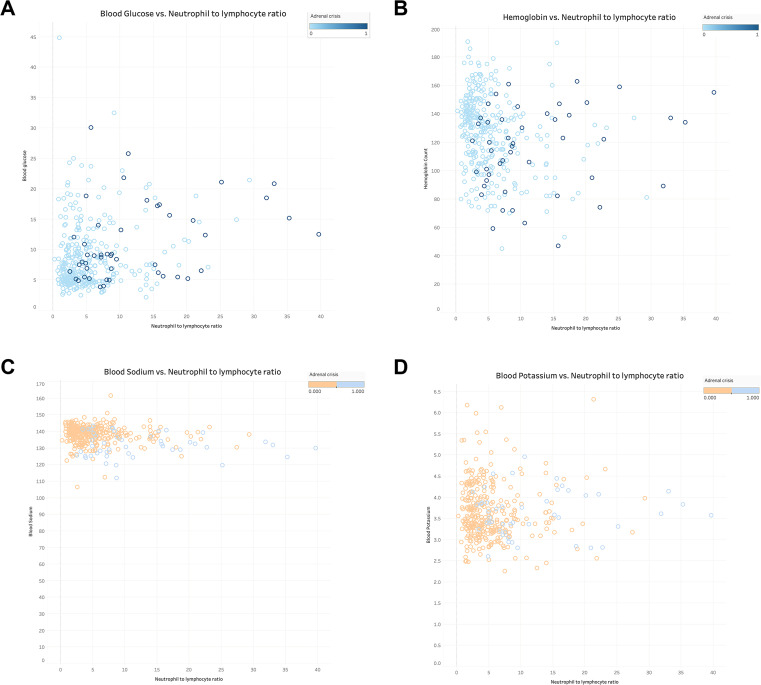
The relationship of neutrophil to lymphocyte ratio with blood glucose, hemoglobin, serum sodium and serum potassium in GIAI patients. This scatter plot shows the relationship between neutrophil to lymphocyte ratio (NLR) and blood glucose **(A)**, hemoglobin **(B)**, sodium **(C)**, and potassium **(D)** levels in GIAI patients, grouped according to the classification of adrenal crises. Each point in the graph represents a patient’s data, with the horizontal axis representing the NLR value and the vertical axis representing the corresponding physiological parameter level. Different colors or markers represent different adrenal crisis states, where 0 indicates that the patient does not have adrenal crisis and 1 indicates that the patient has adrenal crisis.

**Figure 4:**
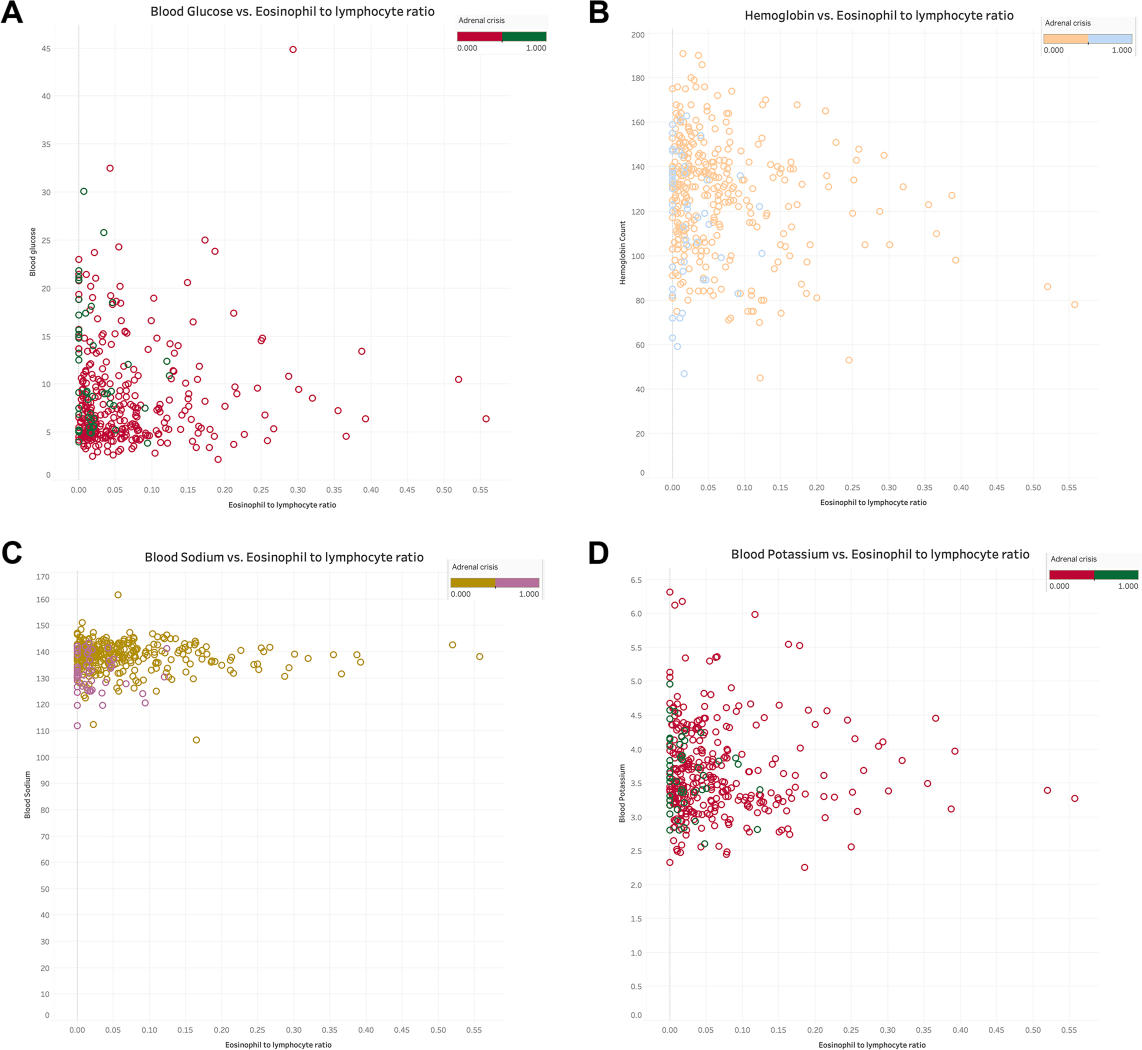
The relationship of eosinophils to lymphocyte ratio with blood glucose, hemoglobin, serum sodium and serum potassium in GIAI patients. This scatter plot shows the relationship between eosinophils to lymphocyte ratio (ELR) and blood glucose **(A)**, hemoglobin **(B)**, sodium **(C)**, and potassium **(D)** levels in GIAI patients, grouped according to the classification of adrenal crises. Each point in the graph represents a patient’s data, with the horizontal axis representing the ELR value and the vertical axis representing the corresponding physiological parameter level. Different colors or markers represent different adrenal crisis states, where 0 indicates that the patient does not have adrenal crisis and 1 indicates that the patient has adrenal crisis.

The relationship between NLR and ELR in GIAI patients and blood glucose, hemoglobin, sodium and potassium were plotted. Where “0” indicates that the patient did not have AC and “1” indicates that the patient had AC. The results shown in **Figures 3(A–D)**, **4(A–D)** showed that patients with higher NLR and lower ELR had higher blood glucose and potassium, lower blood sodium and hemoglobin, and a higher risk of AC.

## 4 Discussion

Infection is an important factor promoting the development of AC. Cytokines released by infection, such as IL-1, TNFα and IL-6, will activate the HPA axis, and the endogenous cortisol level in those with preserved adrenal function will increase significantly at this time to inhibit inflammatory cytokines and reduce inflammatory response (17). However, patients with AI cannot effectively increase glucocorticoid secretion under stress, seriously increasing their risk of AC and even death (18, 19). Patients with PAI are more likely to develop AC than SAI patients, possibly because some SAI patients still retain a certain degree of cortisol secretion capacity (11). However, when the emergency demand for cortisol exceeds its supply in the bloodstream, all AI patients are at risk of AC. This study also found that patients with AC faced a higher risk of coinfection compared to those without AC. On the one hand, many studies have confirmed that AC patients are more susceptible to bacterial infections, possibly due to their reduced immune system function, particularly in GIAI patients treated with exogenous glucocorticoids, which can cause complex immunoregulatory imbalances (14). Their susceptibility to certain bacteria and opportunistic fungi is significantly increased (20). On the other hand, when infection occurs, GIAI patients may have an uncontrolled inflammatory response in the absence of cortisol, leading to serious consequences such as tissue damage, hypotension, shock, and even multiple organ failure.

NLR is widely used in clinical practice. As a novel nonspecific inflammation marker, NLR has shown its unique value in the diagnosis and prognosis prediction of various diseases (21–23). It reveals the relationship between lymphocytes that regulate inflammation and neutrophils that verify activation, and an increase in NLR is generally associated with a more severe inflammatory imbalance and higher inflammation levels (24). Recent studies have shown NLR’s potential in prognosticating adrenal tumor patients, with some indicating that a higher preoperative NLR in patients undergoing adrenal cortical carcinoma (ACC) surgery may predict shorter median overall survival (25). In the present study, patients who developed AC exhibited significantly higher NLR and a higher risk of coinfection than those who did not, suggesting a more severe inflammatory response in the patients. Elevated NLR may indicate insufficient adrenal function in response to inflammation, failing to control the inflammatory process effectively, leading to insufficient hormone secretion and increasing the risk of AC.

ELR is another hematological inflammatory biomarker with predictive value for certain diseases (26). Some studies have found that eosinophilia may serve as a marker of bacterial infection in different types of patient populations (27, 28). Eosinophils play a homeostatic role in the body’s immune response and have been implicated in a variety of autoimmune diseases (29). Although some studies suggest that AC may be accompanied by increased eosinophils, the higher risk of AC in GIAI patients with higher ELR levels, along with a higher number of coinfections in these patients as observed in this study, may indicate an immune system imbalance. The use of GCs by GIAI patients leads to a decrease in immune cells, including eosinophils. When AC occurs due to a sudden and severe deficiency of adrenal hormones, it stimulates the body to transfer eosinophils from the bloodstream into tissues, resulting in further eosinophil depletion and a decrease in the body’s defense against foreign pathogens (30).

In this study, we also found that GIAI patients with psychiatric symptoms, such as anxiety and depression, may be more susceptible to AC. Additionally, psychological and cognitive challenges, as well as social isolation, may impede patients’ capacity to manage their AI. Non-compliance with GC replacement therapy may also significantly increase the risk of AC (9). The onset of AC may be delayed, making it crucial for patients to recognize the body’s stress response, such as during infection, and adjust their drug dosage or administer corticosteroids promptly if needed to prevent AC. However, many patients are skeptical about the need for GC, concerned about its possible side effects, and lack the skills to take action to avoid AC episodes (31).

The research results showed that infection, psychiatric symptoms, serum sodium, albumin, NLR, and ELR were independent risk factors for AC, and these findings were consistent with the results of existing studies on poor prognosis factors in GIAI patients (2, 32). Additionally, in terms of model construction, this study employed several machine learning algorithms, including logistic regression, decision tree, random forest, and SVM, to find the most appropriate model for predicting AC in the GIAI patient dataset. The results showed that logistic model and random forest model had the best predictive effect in this dataset.

Although this study has achieved some results in identifying risk factors for poor prognosis in GIAI patients and constructing predictive models, the study has certain limitations. Firstly, as a retrospective cohort study, there may be selection bias and information bias, which may affect the generalizability of the results. Second, the single-center nature of the study may limit the generalizability of the results. Patients in different regions may have different clinical presentations and outcomes due of differences in genetics, environment, lifestyle, and medical practices. Although 371 GIAI patients were included, the sample size may still be insufficient to detect all potential predictors and risk factors. Future studies should consider using a prospective design, including more comprehensive patient characteristics, conducting multicenter studies, and performing long-term follow-up to validate and enhance the stability and generalization ability of the prediction model.

## 5 Conclusions

In this study, we conducted a comprehensive analysis of the clinical characteristics of patients with GIAI and identified infection, psychiatric symptoms, serum sodium level, albumin level, NLR, and ELR as independent predictors of AC in these patients. Particularly, NLR and ELR emerged as potent indicators for the risk of AC, and we combined them with other clinically significant indicators to construct an effective prediction model. These findings are important references for clinicians when formulating treatment plans and conducting risk assessments. Future studies should further explore the biological mechanisms of these prognostic factors and develop more accurate individualized prediction models.

## Data Availability

The original contributions presented in the study are included in the article/supplementary material. Further inquiries can be directed to the corresponding author.
